# Reactive oxygen species in organ-specific autoimmunity

**DOI:** 10.1007/s13317-016-0083-0

**Published:** 2016-08-04

**Authors:** Giulia Di Dalmazi, Jason Hirshberg, Daniel Lyle, Joudeh B. Freij, Patrizio Caturegli

**Affiliations:** 1 Division of Immunology, Department of Pathology, The Johns Hopkins School of Medicine, Baltimore, MD 21205 USA; 2 Department of Medicine, G. D’Annunzio University of Chieti, Chieti, Italy; 3 Department of Biochemistry and Molecular Biology, The Johns Hopkins School of Public Health, Baltimore, MD USA; 4 Department of Molecular Microbiology and Immunology, The Johns Hopkins School of Public Health, Baltimore, MD USA

**Keywords:** Reactive oxygen species (ROS), Oxidative stress, Autoimmunity, Hashimoto thyroiditis, Monoamine oxidase (MAO), Smoking

## Abstract

Reactive oxygen species (ROS) have been extensively studied in the induction of inflammation and tissue damage, especially as it relates to aging. In more recent years, ROS have been implicated in the pathogenesis of autoimmune diseases. Here, ROS accumulation leads to apoptosis and autoantigen structural changes that result in novel specificities. ROS have been implicated not only in the initiation of the autoimmune response but also in its amplification and spreading to novel epitopes, through the unmasking of cryptic determinants. This review will examine the contribution of ROS to the pathogenesis of four organ specific autoimmune diseases (Hashimoto thyroiditis, inflammatory bowel disease, multiple sclerosis, and vitiligo), and compare it to that of a better characterized systemic autoimmune disease (rheumatoid arthritis). It will also discuss tobacco smoking as an environmental factor endowed with both pro-oxidant and anti-oxidant properties, thus capable of differentially modulating the autoimmune response.

## Introduction

Reactive oxygen species (ROS), also known as free radicals, were first described by Fenton in 1894 [1] and then studied since the mid 1950s for their involvement in aging and age-related conditions [2]. In more recent years, ROS were shown to play a role in physiological processes [3] (such as the synthesis of thyroid hormones and proliferation of thyroid follicular cells [4]), in cellular signaling as second messengers [5], in the normal response of phagocytes to intracellular pathogens, and in a variety of pathological conditions ranging from sarcopenia [6] to cancer [7].

**Fig. 1:**
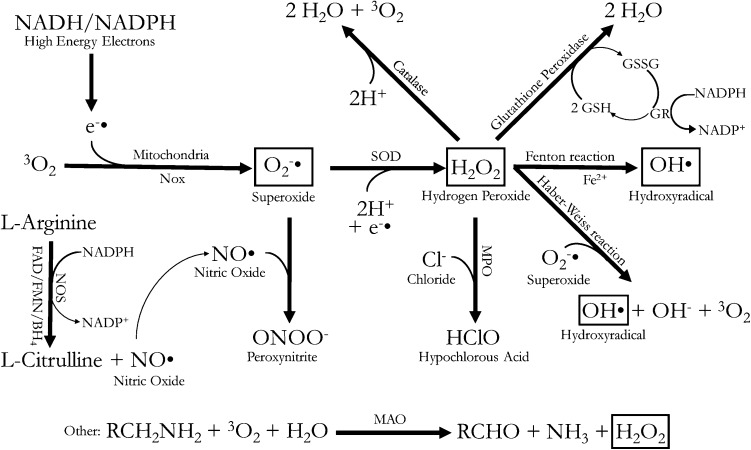
Schematic representation of the three major reactive oxygen species (superoxide, hydrogen peroxide, and hydroxyradical) and the enzymatic pathways that produce them. *NADH* nicotinamide adenine dinucleotide, *NADPH* nicotinamide adenine dinucleotide phosphate, *GSH* glutathione, *GSSG* glutathione disulfide, *SOD* superoxide dismutase, *NOS* nitric oxide synthases, *MAO* monoamine oxidase, *MPO* myeloperoxidase

Oxygen is activated by the addition of electron(s) donated by a variety of substances. This transfer of electrons from a substance (reductant) to another one (oxidant) is called redox reaction, a highly conserved reaction that leads to the production of ROS. There are three major ROS: superoxide anion, hydrogen peroxide, and hydroxyl radical (Fig. 1, boxed).

Addition of one electron to molecular oxygen leads to the production of *superoxide anion* (O_2_^−·^), the precursor of the other two ROS. In fact, superoxide anion can dismutate spontaneously to produce hydrogen peroxide by the addition of another electron and two protons, or be converted enzymatically by the cytosolic superoxide dismutase 1 and the mitochondrial superoxide dismutase 2 (Fig. 1). *Hydrogen peroxide* (H_2_O_2_), a stable ROS, then diffuses through lipid bilayers or intramembranous aquaporins [8], and likely represents the dominant ROS involved in redox signaling due to its stability. Through the classic Fenton reaction (based on the reduction of transition metals, for example from Fe^2+^ to Fe^3+^), H_2_O_2_ is then split into *hydroxyl radical* (OH^·^) and a hydroxide ion. Hydroxyl radicals are highly reactive and exist for only a microsecond, resulting in an oxidative damage that localizes to the site where they are produced [9]. Hydroxyl radicals can also be formed directly from superoxide anion in the presence of hydrogen peroxide through the Haber–Weiss reaction (Fig. 1).

Several additional molecules can be produced from the three main ROS described above. For example, H_2_O_2_ in the presence of a chloride anion is converted by myeloperoxidase into hypochlorous acid, a species important for destroying pathogens within the phagocytic compartment of immune cells [10]. Superoxide can also react with nitric oxide to produce a highly reactive peroxynitrite species (Fig. 1).

This cellular production of ROS is counterbalanced by the presence of numerous molecular and enzymatic antioxidants. Molecules that work as anti-oxidant include vitamins C, A and E, uric acid, glutathione, pycnogenol, and thioredoxin [11]. Antioxidant enzymes include catalase, thioredoxin reductase, glutathione peroxidase, glutathione reductase, glutathione S-transferase, ascorbate peroxidase, ascorbate reductase, and glucose-6-phosphate dehydrogenase [12]. Catalase neutralizes two hydrogen peroxide equivalents into two waters and one molecular oxygen (Fig. 1). On the other hand, glutathione peroxidase uses glutathione as a reducing agent to generate two water equivalents from one hydrogen peroxide species (Fig. 1). To regenerate the pool of glutathione, glutathione reductase converts nicotinamide adenine dinucleotide phosphate to its oxidized form, return oxidized glutathione into its reduced form [13, 14].

## Cellular sources that produce ROS

**Fig. 2:**
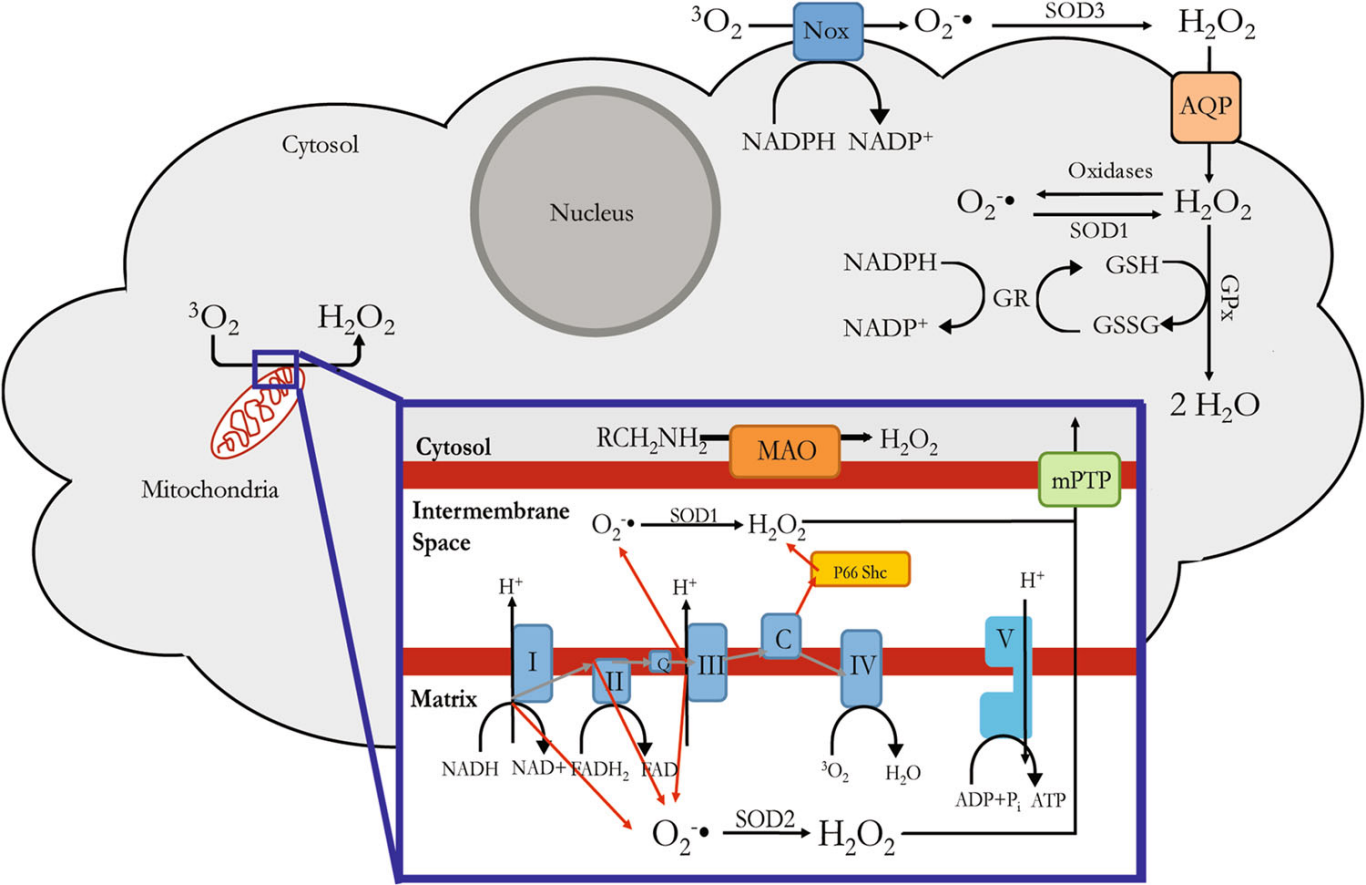
Representation of the main cellular locations where ROS are produced. The abbreviations are the same as those used in Fig. 1, plus the following: *Nox* non-phagocytic NADPH oxidase, *AQP* aquaporin, *GPx* glutathione peroxidase, *GR* glutathione reductase, *mPTP* mitochondrial permeability transition pore, *FAD* flavin adenine dinucleotide, *FADH2* flavin adenine dinucleotide

There are three major intracellular sources of ROS: electron leak from the mitochondrial respiratory chain, NADPH oxidases, and uncoupled nitric oxide synthase reactions (Fig. 2). ROS can also be generated by monoamine oxidase, and other oxidases such as xanthine oxidase, lipoxygenases, cyclooxygenases, and monooxygenases.

### Electron leak from the mitochondrial respiratory chain

Mitochondria generate about 90 % of all ROS [15] during the process of ATP production. This process, called oxidative phosphorylation, is driven by the electron transport chain, which consists of five protein complexes located on the inner mitochondrial membrane (Fig. 2, box). The first four complexes utilize oxygen and high-energy electrons to generate a proton gradient in the intermembrane space. The gradient then provides the energy needed to drive the production of ATP by complex. During cellular stress, electrons leak from the respiratory chain and react with molecular oxygen to generate superoxide anion and the secondary ROS [15], which then leave the mitochondria through the permeability transition pore located on the outer membrane [16].

Complex 1, 2, and 3 are the primary sites for ROS production [17], although other factors such as the ratio of ubiquinol to ubiquinone, the mitochondrial membrane potential, and the proton-motive force may be involved [15]. Complex 1 can reduce oxygen to superoxide on the leaflet facing the mitochondrial matrix, and contains a Q-binding site, a flavin mononucleotide, and multiple iron-sulfur clusters that directly participate in ROS production [15, 18]. Complex 2 can alter its catalytic activity to modulate directionality of the electron transport chain to promote ROS production [19], especially when complex 1 or 3 is impaired [20]. Alternative sources of mitochondrial ROS may result from cytochrome C electron transfer to p66-Shc, which subsequently transfers electrons to oxygen in the intermembrane space to generate both superoxide and hydrogen peroxide [21]. Complex 3 transfers electrons from ubiquinol to cytochrome C and mostly generates ROS at the Q-binding site, which diffuses into both the matrix and intermembrane space [15].

### NADPH oxidase

Nicotinamide adenine dinucleotide phosphate (NADPH) oxidase is a flavocytochrome originally discovered in phagocytes. Its function is to release superoxide or hydrogen peroxide in the phagocytic compartment to neutralize pathogens. NADPH oxidase generates superoxide by the catalytic transfer of high energy electrons from the nicotinamide moiety of NADPH to flavin adenine dinucleotide, and then to molecular oxygen.

Phagocytic NADPH oxidase is an assembly of membrane-bound p22 oxidase (p22^phox^), gp91^phox^ catalytic subunit, and at least four other cytosolic subunits: p47^phox^, p40^phox^, p67^phox^, and a small G-protein Rac or p21 Rac [22]. Non-phagocytic NADPH oxidase (abbreviated as Nox) refers to homologues of the catalytic gp91 subunit found in non-phagocytic cells [23]. They have similar structure to the phagocytic NAPDH but different biological functions. They are found in endothelial cells and fibroblasts and respond to pro-inflammatory cytokines such as tumor necrosis factor alpha by enhancing superoxide production [24–27], although less efficiently than the phagocytic form. There are seven Nox isotypes (Nox 1 through 5, dual oxidase 1, and dual oxidase 2), mostly localized to the plasma membrane [28]. They generate ROS on the cytosolic leaflet of the plasma membrane or release them into the extracellular milieu, as Nox-containing vesicles fuse with the plasma membrane during Nox activation [28]. ROS produced in this fashion can in turn inhibit Nox to maintain a low basal oxidative state or upregulate it in a feed-forward mechanism [13, 29, 30]. Nox-derived ROS are involved in apoptosis and fibrosis of various tissues [31].

### Uncoupled nitric oxide synthase reactions

Nitric oxide synthase (NOS) converts l-arginine into citrulline, using tetra-hydro-biopterin as cofactor, as well as other substrates. This reaction releases nitric oxide (NO^·^) (Fig. 1), an important gas that mediates vasodilation and is involved in numerous other functions, such as the immune response against parasites [32]. There are three main types of NOS: neuronal, endothelial, and inducible. If substrates become limiting or unusable, the reaction uncouples generating large amounts of superoxide, which then reacts with nitric oxide to generate peroxynitrite [10] (Fig. 1), a substance that negates the important vasodilatory effect of nitric oxide. ROS can damage nitric oxide synthase and induce more ROS production in a feed-forward mechanism fashion, promoting in this case endothelial apoptosis, hyper coagulation, and monocyte adhesion [33].

### Monoamine oxidase

Monoamine oxidase (MAO) is located on the cytosolic leaflet of the outer membrane of mitochondria and mediates the catabolism of monoamine neurotransmitters. There are two well-characterized isotypes of MAO that differ because of their substrate specificity: MAO-A predominantly catabolizes serotonin and noradrenaline, while MAO-B preferentially deaminates phenylethylamine and benzylamine. Dopamine and tyramine are metabolized similarly by both MAO isoforms. MAO isoforms are expressed in several other tissues besides the nervous system: cardiomyocytes, hepatocytes, duodenal villi, vessels, renal collecting tubules, and Bowman’s capsule [34]. In the pancreatic islets, MAO-B is expressed in beta and alpha cells and negatively regulates insulin secretion [35]. MAO contribute to ROS production mainly through the synthesis of H_2_O_2_ [36] (Fig. 2).

Inhibition of MAO, and thus inhibition of neurotransmitter degradation, represented the first pharmacological treatment developed for depression in the early 1950s. More recently, MAO inhibitors are being used in other conditions because of their ability to decrease ROS production. For example, Kalurdercic and colleagues identified MAO-derived hydrogen peroxide as a contributor to cardiac damage in ischemia reperfusion injury, and proposed MAO inhibitors as a treatment for this condition [37]. Recently, MAO-derived ROS have been linked to cardiomyocyte necrosis and heart failure by impairing activation of transcription factor-EC activation and mitochondria clearance by lysosomes [38]. In the field of autoimmunity, it has been shown that the MAO inhibitor phenelzine ameliorates disease outcomes in a mouse model of multiple sclerosis [39]. Furthermore, phenytoin, an anticonvulsant that inhibits norepinephrine release and MAO activity, induces proliferation in cultured melanocytes and has therefore, been proposed as a treatment for vitiligo [40].

## ROS in autoimmune diseases

The role of ROS in autoimmunity is complex. The traditional view holds that ROS accumulation is detrimental to the autoimmune disease process. Oxidative stress ensues when the production of ROS surpasses the buffering capacity of the endogenous antioxidants [41], leading to oxidation of lipids in the plasma membrane, proteins in cytosol and nucleus, and nucleic acids that overall damage the cells in the organ targeted by autoimmunity. Oxidative stress can also lead to the generation of novel autoantigens and thus exacerbation of the autoimmune response [42]. In keeping with these findings, ROS production has been linked directly to inflammation via the production of TNF-α: Salzano et al. reported that macrophages release an oxioreductase that directly stimulates TNF-α [43]. But more recent studies reveal a regulatory role of ROS where they prevent progression of chronic inflammatory responses (reviewed in [44]).

Two excellent reviews have been published on the role of ROS in systemic autoimmune and inflammatory diseases [45, 46]. We will discuss here the contribution of ROS to four organ-specific autoimmune diseases, comparing it to that reported in rheumatoid arthritis, and use tobacco smoking as an example of an environmental factor that can function both as an oxidant and anti-oxidant.

### ROS and Hashimoto thyroiditis

As mentioned in the introduction, ROS are fundamental for the normal functioning of the thyroid follicular cell. ROS, however, have also been implicated in the pathogenesis of Hashimoto thyroiditis, in both murine and human models.

The NOD-H2^h4^ mouse is a congenic strain that develops autoimmune thyroiditis spontaneously but at a low incidence, an incidence that can, however, be significantly increased by addition of iodine to the drinking water [47, 48]. Burek and colleagues showed that thyrocytes isolated from NOD-H2^h4^ mice produced significantly more H_2_O_2_ than control thyrocytes when exposed to iodine [49]. They also associated this increased ROS load with higher expression of intracellular adhesion molecule-1 on thyrocytes [50], and therefore, with greater retention capacity of the lymphocytes that infiltrate the thyroid gland. Incubation with the antioxidant diphenyleneiodium, an inhibitor of NADPH oxidase, reduced ROS generation and adhesion molecule expression in cultured NOD-H2^h4^ thyrocytes [50]. Kolypetri and Carayanniotis showed that ROS increase the apoptosis of NOD-H2^h4^ thyrocytes exposed to iodine [51]. Thyroidal accumulation of ROS has also been shown to promote cleavage of thyroglobulin into several fragments, likely exposing the immune system to novel epitopes and thus enhancing the autoimmune response [52]. Finally, increased content of 4-HNE, a toxic product from lipid peroxidation used as a marker of oxidative stress, was found in NOD.H2^h4^ thyroid glands [53]. Overall, studies in the NOD.H2^h4^ model suggest that thyroidal accumulation of ROS contributes to the initiation and progression of autoimmune thyroiditis.

Studies in patients with Hashimoto thyroiditis are more limited. Ates and colleagues compared 93 cases with Hashimoto thyroiditis (a third in each of the euthyroid, subclinical hypothyroidism, and overt hypothyroidism subgroups), to 31 healthy controls. They found that oxidative stress parameters in the peripheral blood were higher in cases than controls, particularly in the overt hypothyroidism group [54]. In a smaller study of 35 euthyroid Hashimoto cases and 35 healthy controls, Baser et al. reported that serum oxidant status was higher in cases than controls, and positively correlated with the levels of thyroglobulin antibodies [55]. Finally, Ruggieri et al. analyzed 71 euthyroid Hashimoto thyroiditis cases and 63 healthy controls, reporting that oxidative stress is higher in cases and that thyroperoxidase antibodies are the main predictor of the oxidative status independent of thyroid function [56]. Overall, human studies report an increased oxidative status in Hashimoto thyroiditis but do not clarify whether this is the cause or result of thyroid dysfunction.

### ROS and inflammatory bowel disease

Increased levels of ROS have been reported throughout the colon of patients with inflammatory bowel disease. The increase is not limited to areas of active inflammation, thus suggesting a role for oxidative stress during the early phases of disease pathogenesis [57]. Interestingly, because of the high rate of depression in inflammatory bowel disease, MAO inhibitors are commonly prescribed in this patient population [58]. In a review of studies examining the effectiveness of antidepressants, the MAO inhibitor phenelzine improved both psychiatric and somatic symptoms of inflammatory bowel disease [59]. In addition, numerous case reports have documented clinical improvement or remission of the bowel inflammation upon usage of phenelzine [59, 60]. The normal colon is the organ that, after the placenta, expresses the highest levels of MAO-A, and it is reasonable to postulate that these levels increase even further upon inflammation. Indeed, Magro and colleagues identified markedly lower levels of monoamines in the mucosa of ulcerative colitis patients, suggesting MAO hyperactivity and consequently increased ROS production [61]. Although no study has directly assessed the link between MAO activity and ROS levels in gut mucosa, or their temporal relationship with bowel inflammation, we suggest that MAO inhibition exerts its beneficial effects in ulcerative colitis by lowering the levels of ROS.

### ROS and multiple sclerosis

Given the high levels of oxidative activity found in neurological tissues, it is not surprising that ROS have long been associated with the pathological damage typical of multiple sclerosis [62]. Indeed, several oxidized molecules can potentially be used as diagnostic biomarkers [63]. Increased levels of peroxynitrite are found in active multiple sclerosis lesions [64]. ROS have also been implicated in the dysregulation of the blood–brain barrier, which results in faster disease progression due to increased monocyte infiltration and inflammation [65]. By proteomics, Fiorini and colleagues have shown that patients with the relapsing-remitting form of multiple sclerosis have higher levels of oxidized proteins than healthy controls [66]. Ceruloplasmin, antithrombin III, clusterin, apolipoprotein E, and complement C3, were upregulated in cases; vitamin D-binding protein showed an increasing trend toward oxidation in patients going from remission to relapse. Using a whole-genome microarray approach, Fischer et al. found that several mitochondrial genes involved in inducing oxidative stress were upregulated in multiple sclerosis patients [67]. They also found that microglial cells and astrocytes upregulated the p22 subunit of the Nox2 complex within active pathological lesions. These findings suggest that ROS are involved in early disease stages of multiple sclerosis, when myelin sheaths are still intact but there is lymphocytic infiltration and microglial activation [65, 67].

### ROS and vitiligo

In patients with vitiligo the epidermis contains increased levels of ROS, mainly H_2_O_2_ and peroxynitrite, as well as inadequate antioxidant defenses [68]. This ROS increase originates from several sources, both exogenous (ultraviolet radiations, trauma, stress, infections, malignancies, certain drugs) and endogenous. First, there is an elevated activity of NADPH oxidase and NOS, with secondary increase production of ROS and reactive nitrogen species [69] (Fig. 1). Then, there is an accumulation of tetra-hydro-biopterin, an essential cofactor for the aromatic amino acid hydroxylases and NOS. Increased biopterin levels boost the formation of H_2_O_2_ and inhibit the function of phenylalanine and tyrosine hydroxylases, thus impairing melanin production in melanocytes and inducing norepinephrine accumulation in keratinocytes [70]. Finally, there is an increased activity of MAO-A, which leads to the accumulation of toxic levels of H_2_O_2_ [71], and a largely impaired mitochondrial function [72]. Low levels of antioxidants, such as catalase, glutathione peroxidase, glucose-6-phosphate dehydrogenase, superoxide dismutase, and vitamins C and E have been reported in the epidermis and serum of vitiligo patients [73–75]. Recently, Jian and colleagues have shown that the anti-oxidant response element nuclear factor E2 protects melanocytes from H_2_O_2_ damage through the induction of antioxidant genes, such as heme oxygenase-1, and that this pathway is functionally deficient in vitiligo melanocytes, rendering them more susceptible to oxidative stress [76, 77].

Increased skin content of ROS not only directly damages the melanocytes but also induces an autoimmune response against them. In fact, ROS modify the structure of key vitiligo autoantigens such as melan A and tyrosinase, leading to the formation of novel epitopes which then trigger autoreactivity. During the early stages of vitiligo, lipid peroxidation levels, a marker of oxidative stress, have been reported to be increased, whereas melanocyte antibodies appear in later disease stages [72], suggesting that ROS play a role in initiating vitiligo and amplifying the loss of melanocytes.

### ROS and rheumatoid arthritis

Oxidative stress plays an important role in the pathogenesis of rheumatoid arthritis [78]. Staroń and colleagues analyzed erythrocytes from rheumatoid arthritis cases and healthy controls and reported increased lipid peroxidation, decreased activity of antioxidant enzymes, and decreased sodium–potassium ATPase functions [79]. In the synovial cavity, Mapp et al. have found increased ROS content, leading to the oxidation of immunoglobulins, mainly IgM, recognizing the Fc portion of IgG (so called, rheumatoid factor), lipoproteins, lipids, and hyaluronan [80]. Immunoglobulins damaged by oxidation are more sensitive to non-enzymatic degradation by sugars, primarily at arginine and lysine residues, leading to the formation of advanced glycation end products [78], as it is seen in diabetes mellitus where prolonged hyperglycemia glycoxidates hemoglobin into hemoglobin A1c. Indeed, antibodies to glycoxidized IgG are specifically found in patients with early synovitis [81]. Increased ROS also damage the DNA mismatch repair system, which is defective in rheumatoid arthritis, and the DNA itself, resulting in elevated concentrations of 8-oxo-7-hydro-deoxyguanosine [78]. Further tissue damage originates from ROS produced by monocytes and neutrophils [82]. Neutrophils degranulate in the synovial joint releasing myeloperoxidase that, using chloride and H_2_O_2_, catalyzes the formation of hypochlorous acid (Fig. 1). Hypochlorous acid is a very strong oxidant: it mainly reacts with methionines and cysteines disrupt protein tertiary structure and activity [83]. In keeping with a pathogenic role of ROS, rheumatoid arthritis patients who improve upon treatment with monoclonal antibodies that block tumor necrosis factor alpha do show reduced plasma levels of ROS [84].

Animal models of rheumatoid arthritis have been used to assess the potential therapeutic benefits of anti-oxidant administration. In the adjuvant-induced rat model of the disease, paeoniflorin significantly improved the arthritic symptoms and increased the pain threshold, changes that were associated with boosted activity of the anti-oxidant enzymes catalase and glutathione peroxidase [85]. In the collagen-induced rat model of arthritis, administration of the antioxidant thymoquinone improved arthritis scoring and bone histopathology while reducing pro-inflammatory cytokines and ROS content [86].

## Tobacco smoke as a modulator of the oxidants/anti-oxidants balance

Tobacco smoke contains a variety of ROS, reactive nitrogen species, and other compounds that increase the burden of oxidative stress [87]. It is unquestionable that smoking has deleterious effects on human health, most notably related to a higher risk of chronic respiratory diseases [77, 88, 89] and cancer [90, 91]. Intriguingly, however, smoking can also be beneficial in a selected group of conditions, for example in patients with ulcerative colitis [92–95], Hashimoto thyroiditis (see Supplemental Table 1 in [96]), and Parkinson disease [97, 98]. In addition, it is known that small amounts of ROS protect the myocardium from hypoxic damage, in a process termed ischemic preconditioning [99]. It is thus possible that ROS acquired from cigarette smoking precondition organs targeted by autoimmunity by inducing some measures of protection. An excellent review on the varied effect of smoking in autoimmune diseases has been recently published [100]. Here, we will focus on the pro-oxidant and anti-oxidant effects of smoking, using autoimmune thyroiditis as an example.

### Pro-oxidant effects of smoking

ROS in smoking cause oxidative damage to DNA, as indicated by increased urinary levels of 8-hydroxy-2′-deoxyguanosine [101]. Similarly, bronchoalveolar lavage fluid levels of this molecule positively correlate with smoking status [102]. Smoking also contains reactive nitrogen species [77] and thiocyanate that, despite operating through different mechanisms, also leads to increased oxidation in certain organs. Thiocyanate, in fact, competitively inhibits the uptake of iodine by the sodium-iodide symporter, thus reducing the concentration of iodine inside the thyroid cell and possibly leading to higher oxidative load [103].

Some carcinogens generated during the combustion of tobacco contribute to oxidation. For example, 2-amino-9*H*-pyrido [2,3-*b*]indole (abbreviated as AαC) becomes activated in vivo to form N-oxidized metabolites that covalently bind to DNA (DNA adducts) and albumin (albumin adducts), promoting mutations and loss of function [104]. Normal albumin, the most abundant protein in human serum, normally serves as an anti-oxidant because it scavenges ROS generated during cell metabolism or introduced from the environment. Albumin adducts loose this protective property.

### Anti-oxidant effects of smoking

Tobacco leaves contain compounds that inhibit the activity of MAO, and thus reduce the amount of ROS produced by these enzymes. Smokers are known to have lower MAO activity than non-smokers. For example, positron emission tomography brain scans using ^11^C-based tracers that bind to catalytically active MAO have demonstrated a reduced MAO activity in smokers [105, 106]. More specifically, [^11^C]clorgyline, a potent and irreversible inhibitor of MAO-A, showed a mean 28 % reduction in MAO-A activity (ranging from 22 to 38 %) across all cortical and subcortical regions imaged [105]. Similarly, using [^11^C]befloxatone, which also binds MAO-A reversibly, there was a 60 % average reduction in smokers in cortical regions [106]. Different tracers or earlier scans (within 2 h from the last cigarette smoked) could explain the different percent inhibitions observed in the two studies [107].

What are the compounds in cigarette smoking that inhibit the activity of MAOs? Likely many and their presence is perhaps a reason why smokers have difficulty quitting: MAO inhibition, in fact, could provide a pleasant anti-depressant effect, although no solid data support this hypothesis [108]. Trans–trans-farnesol and 2,3,6-trimethyl-1,4-naphthoquinone specifically inhibit MAO-B [109]; β-carboline alkaloids inhibit MAO-A and MAO-B [106]. Nicotine, the major tobacco alkaloid, could also have MAO inhibitory properties. Recently, it has been in fact shown that nicotine chelates ferrous ion (Fe2^+^) in a concentration-dependent fashion. Since Fe2^+^ can produce ^·^OH through the Fenton reaction (Fig. 1), these results support a role for nicotine and related alkaloids as antioxidants [110].

Collectively, these results demonstrate that in the highly complex mixture of chemicals characteristic of tobacco smoke there are compounds that have pro-oxidant, antioxidant, or both kinds of properties. Future studies that examining the oxidation status globally, rather than selectively focusing on oxidative or antioxidant effects, could help unravel the overall role of smoking tobacco with regard to oxidation.

## Conclusions

The role of ROS in autoimmunity remains complex. ROS accumulation has been implicated both in the initiation and progression of autoimmunity, but is still unclear whether it represents a *bona fide* trigger or a harmless accompaniment. It is, however, intriguing to consider the development of selective ROS inhibitors as a tool that could be used to treat a broad spectrum of autoimmune diseases.

## Abbreviations

ROS: Reactive oxygen species
NADPH: Nicotinamide adenine dinucleotide phosphate
NOS: Nitric oxide synthase
MAO: Monoamine oxidase
